# The second patient? Family members of cancer patients and their role in end-of-life decision making

**DOI:** 10.1186/s12904-018-0288-2

**Published:** 2018-02-17

**Authors:** Katsiaryna Laryionava, Timo A. Pfeil, Mareike Dietrich, Stella Reiter-Theil, Wolfgang Hiddemann, Eva C. Winkler

**Affiliations:** 1 National Center for Tumor Diseases, Department of Medical Oncology, Programme for Ethics and Patient-Oriented Care in Oncology, Heidelberg University Hospital, Im Neuenheimer Feld 460, 69120 Heidelberg, Germany; 2 Department of Internal Medicine III (Haematology and Oncology), University Hospital Grosshadern, Munich, Germany; 3 Department Clinical Ethics, Psychiatric Hospitals of the University Basel, University Hospital Basel, University of Basel, Basel, Switzerland

**Keywords:** Medical oncology, Medical ethics, Communication, Palliative care, End-of-life decisions, Family members, Qualitative research

## Abstract

**Background:**

Family members are important companions of severely ill patients with cancer. However, studies about the desirability and difficulties of integrating relatives in the decision-making process are rare in oncology. This qualitative study explores the family role in decisions to limit treatment near the end of life from the professionals’ point of view.

**Methods:**

Qualitative in-depth interviews were conducted with oncologists (*n* = 12) and nurses (*n* = 6) working at the Department of Hematology/Oncology at the University Hospital in Munich, Germany. The data were analyzed using a descriptive qualitative methodology and discussed from a medical ethics perspective.

**Results:**

Four major themes played a central role in the perception of the medical staff in regard to family members. (1) Family impact on patients’ treatment preferences. (2) Strong family wish for further treatment. (3) Emotional distress of the family related to the involvement in end-of-life decision-making. (4) Importance of knowing family structures.

**Conclusions:**

The important role of the family members in patients’ disease process is recognized by oncologists and oncology nurses. However, this does not seem to lead to an early involvement of the family members. Developing and establishing a systematic assessment of family members’ needs and wishes in order to provide a specific-tailored support should become a priority for interdisciplinary clinical research in the near future.

## Background

Family members are important companions and an irreplaceable source of support for severely ill patients near the end of life [1–8]. They provide emotional support for patients and play a considerable role in patients’ choice of a treating oncologist, hospital, and in decisions over possible treatment options. Furthermore, family can facilitate or hinder a patient’s decision against further cancer-specific treatment in favor of best supportive care (BSC) with a focus on symptom control and quality-of-life [9, 10].

A qualitative study with 37 patients with advanced cancer and40 caregivers conducted by Zhang et al. showed that 65% of families had disagreements regarding treatment decisions including discontinuation of anti-cancer treatment, thus considerably influencing patients’ treatment choices [11].

Empirical evidence demonstrates that patients place a great importance on communication regarding treatment options within their family [12]. In a study by Schäfer et al. the majority (89%) of the surveyed oncological patients preferred medical decisions to be made jointly with their family and oncologists [13, 14]. In a study by Hobbs et al. 49.4% of 5204 surveyed patients with lung and colorectal cancer reported that they would involve family members in decision making [15].

In a cross-sectional interview study with 130 patients who were diagnosed with incurable disease, Nolan et al. showed that if patients were unable to decide for themselves in case of a severe illness, patients would feel better if they could be represented by their relatives, rather than by their doctors [16].

It has also been shown, that family involvement in decisions about forgoing treatment has positive effects such as reducing patients’ distress and anxiety and increasing their quality of life [17]. A study conducted by Dionne-Odom et al. with 122 caregivers demonstrated that family members who were integrated quite early showed lower depression and distress scores [18].

Additionally, timely involvement of family members at different stages of decision-making process may help family members to adjust to patients’ incurable disease, to become more prepared for the approaching death and to reduce their psychological distress [3, 19]. In clinical practice, however, family members are oftentimes involved in decision-making only to a small extent [20]. In a study on decision-making in the Intensive Care Unit (ICU), Azoulay et al. showed that even though the majority (91%) of physicians hold the opinion that family members should be involved in treatment decisions for patients without decision-making capacity, only in one third of patients’ cases family members were in fact involved in decision-making [21]. It is a similar situation with patients who are capable of making decisions: only in one-third of patients’ cases family members were actually involved in treatment decisions [2].

The previous research has mostly focused on a family role in decisions regarding treatment options [22–27]. However, studies about the desirability and difficulties of integrating relatives in the decision-making regarding treatment limitations near the end of life and switching to BSC from the professionals’ point of view are missing in oncology, where patients are often capable of making decisions to the end.

Therefore, we conducted a qualitative interview study with oncologists and oncology nurses in order to gain in-depth understanding of the family role in decision-making for the specific context of a treatment limitation. These interviews focus on forgoing cancer specific treatment, but also touch on topics such as referral to ICU and “Do not resuscitate order”. The focus of our study was on the family role in decision- making when the patient is able to communicate and make decisions. Our aim was to identify and illuminate oncologists’ rationales for and against family involvement as well as challenges perceived by oncologists and oncology nurses while involving family members in the decisional process of a treatment limitation.

## Methods

### Study design and participants

A descriptive qualitative research approach - as Sandelowski [28] described it - was used in order to get information from oncologists and oncology nurses on family involvement in decisions to limit treatment. This methodological approach is especially suited to depict precisely participants’ views and obtain answers to research questions that are of a special relevance to interviewees [28]. We aimed to provide a detailed description of oncologists’ and oncology nurses’ experience in involving family in decision-making process on limiting treatment near the end of life when patients are capable to communicate.

Semi-structured qualitative interviews were conducted with 18 participants in the Department of Internal Medicine, which runs a comprehensive cancer center, at the University of Munich Medical Center in Germany.

### Sampling strategy and developing of the interview guide

We used a purposive sampling strategy to collect our data. Selected participants were first contacted by email with a detailed study description and were re-contacted after 1 week by telephone and invited to participate in the interview study.

For our first interviews we developed a preliminary interview guide. It was developed in three steps: 1) in the first step, review of the existing literature was conducted; 2) in the second step, generated questions were discussed in an interdisciplinary team meeting; 3) the interview guide was discussed with the experts in oncology and social sciences. It included open-ended questions about end-of-life communication with patients and family members and their role in decision-making process.

The preliminary interview guide included such questions as:

- Tell me about your experience of decision-making on treatment limitation by patients with advanced cancer near the end of life;
- What role do the family play in these decisions?
- What is your opinion on family involvement in decision-making to limit treatment near the end of life?
- What challenges do you face by family involvement in decisions to limit treatment?

These questions served to start the conversation and were followed up by going into more detailed discussion.

After completing 8 interviews with oncologists and analyzing our first data, more participants needed to be selected to clarify the emerging themes.

The interview guide was slightly modified and included questions about the best way to involve relatives into decision-making (i.e. What is the best way to integrate relatives in decision-making to limit treatment? What factors might hinder family involvement?). For this reason, we approached oncologists with a special expertise in palliative care and in communication with family members.

Furthermore, we modified questions in order to interview oncology nurses too as they could provide valuable insight into relatives’ involvement and, according to interviewed oncologists, often turned out to be a contact person for families at the care unit. N6 interviews with oncology nurses and with 4 oncologists were added to the data.

A total of 18 participants – oncologists (4 male and 8 female, aged 30–65 years) and nurses (all female, aged 30-50 years) with different working experience and position (fellows and seniors) were recruited. All participants work at 4 general cancer wards caring for hematological and oncological in-patients.

Interviews lasted for 30-100 min. Demographic data was collected at the end of the interview. The demographic characteristics of participants are presented in Table 1.

**Table 1:** Demographic characteristic of participants

Nurses	6
Male	0
Female	6
Age range	30-50 years old
Working experience range	12–20 years
Oncologists	12
Male	4
Female	8
Age range	30-65 years old
Working experience range	9 months to 16 years

### Memo-writing

During the whole research process, we wrote both descriptive and reflective case-based memos on the interviewing process, documented participants’ reactions to the asked questions and also documented our emerging new ideas during interviews. Data from the memos were integrated into analysis.

### Data analysis

Interviews were audiotaped, transcribed verbatim, and analyzed according to the qualitative inductive content analysis, including open coding, creating categories and themes. In the first step of open codings, interview data were analyzed “sentence by sentence”, segmented and open codes were created. A code is understood as a meaningful label for the text that expresses the data contents. Through constant comparison, emerging codes were connected by their similarities to categories.

The relationships between the categories were then analyzed through constant comparison and linked into themes.

As a last step, we explored an intercoder reliability in order to judge the measurement consistency and to achieve validation of the emerged codes. This means that we tested the codes within three independent coders (KL, EW, TP) which assigned the data with the same codes [29]. Analysis was managed in using the data analysis software MAXQDA (VERBI GmbH, Berlin, Germany).

The results were evaluated against the background of the current medical ethics discussion on end-of-life decision-making [30, 31].

### Rigor

The following techniques were used to assess credibility, transparency, analysability and usefulness of the data [32].

#### Credibility

The interviewer was not an employee of the hospital where the study was conducted to guarantee neutrality. However, the interviewer had experience in working with oncological patients as well as expertise in qualitative research methods. Researchers’ participation in interviews and in data interpretation were constantly reflected to reduce personal bias in research. For this reason, field notes were written in which emerging thoughts during the research process were documented. Furthermore, regular interdisciplinary team meetings served as a platform for reflection and acknowledgement of researchers’ previous experience and background on a theory development process.

#### Analysability

All interviews were digitally recorded, transcribed verbatim and checked by team members. After each interview we wrote case-based memos that helped us to analyse data. To increase analysability researcher triangulation was used. Through regular meetings of the interdisciplinary team, with expertise in oncology, social science, and medical ethics, multiple researchers were involved in the analytical process. Emerging discrepancies of the analysis were discussed in order to increase the validity and reliability of the study.

#### Transparency

We discussed results in the context of current research as well as limitations of the study and how they may impact the results.

#### Usefulness

Interview participants were selected with a maximum variance in working experience, positions and age in order to increase the representatives of all aspects of the topic in terms of participants [33]. For our research question it was important to search for the maximum variation in perspectives, ranging from the experienced oncologists to nurses working at 4 hospital units.

## Results

When asked about family role in decision-making to limit treatment near the end of life, interviewed oncologists and nurses often assigned an important role to patients’ family: “I think, they play a very important role.” [Nurse 4] or even as one nurse stated: “Sometimes, they play an even more important role than the patient does” [Nurse 2]. Participants underlined the importance of families in providing treatment advice to the patients: “I think, [they play] an advisory role […]” “They just discuss it and see what is the best.” [Nurse 4] as well as in providing support and care for the patient throughout the disease.

However, they recognized that relatives often felt unprepared for end of life decision-making: “It is so, that partly, relatives have not yet confronted themselves with the situation, that has been known for a long time” [Nurse 4], helpless or overwhelmed and needed psychological support when the patient was dying:

“Relatives cannot stand that you leave the room, and when you are out of the room they start to call for you, because they are uncertain and they are afraid. They rather want that someone is there when he (the patient) is on the verge or if he dies then.” [Nurse 3]

However, when asked about family involvement in decisions, respondents commonly held the opinion that relatives were for the most part not actively involved as long as the patient was able to communicate. The interviewees made it clear that their first choice and contact was always the patient who was capable of making decisions: “The priority in my decision-making has always been the patient as long as he is in full position of emotional and mental force. I listen to what relatives say always merely as a secondary opinion” [Physician 8].

When asked about what was the best way to integrate family members in decision-making, some oncologists saw the necessity to prepare family members slowly for the approaching situation of a patient’s death and suggested that it would be important to create a “humane environment” [Physician 10] and involve family members step by step in the end of life decision-making: “[...] That you not only have the conversation with the patient and family members together, but also that you discuss it with the family-members to prepare them for the situation.” [Physician 3].

Relatives were often compared by oncologists and nurses with second patients. They were perceived not only as patients’ informants and supporters but also as affected by a crisis situation due to a life-threatening disease of their beloved one. Therefore, they needed to be taken care of too. “Also, from the side of physicians and I think from the side of nurses it is important to take care of relatives just like of patients, like a sick person” [Nurse 3].

However, they pointed out some several important aspects that should be taken into consideration when involving family and that might even hinder the family involvement in decision-making: 1) family impact on patients’ treatment preferences; 2) a strong family wish for further treatment; 3) emotional distress of the family related to the involvement in end-of-life decision-making; 4) importance of knowing family structures. These aspects are presented below with appropriate participants’ citations.

### Family impact on patients’ treatment preferences

Oncologists and nurses reported that family oftentimes served as an important advisor for the patient regarding treatment options and that their impact on patients’ preferences might be underestimated:

“Uh, we probably underestimate this a little, because […] the process of discussion goes on even further at home with the family, which of course has an impact on patients.” [Physician 10]

Interviewees also noticed, that patients might strive for a life-prolonging treatment for the sake of the family:

“Well, I think relatives and the whole family have a significant impact. So, I guess, if there is an intact family, the patient just wants everything to be done to have simply more time with his family [...].” “But especially we experience it again and again with young families, where patients do everything only in order to think that they have maybe one chance more to survive.” [Nurse 5]

Patients could be pressured or coerced by their families into making medical decisions that are not in line with their wishes.

### Strong family wish for further treatment

When asked about challenging situations in discussing treatment limitations with family members, a situation that was often perceived as challenging by oncologists and nurses was when family members could not accept patients’ situation:

“Relatives just cannot support it or accept that patient does not want to have it [treatment] now.” [Nurse 4]

In such cases relatives demanded a more intensive treatment than the medical team would support:

“Well, […] but there are always cases where relatives just come up to you and say, that everything should be done.” [Physician 2]

In a case of very demanding family members oncologists involved family in decision-making to protect themselves from possible legal consequences. They admitted that they would seek a conversation with patients’ family even if they had already decided upon treatment limitations in order to prevent any legal action from the family side:

“And of course, for the reason that you want to protect yourself from legal actions, I admit that openly. Frequently, I think if we say that he will not be resuscitated or he’ll not go to the ICU in case of deterioration: these are often things that you might medically justify, however you also protect yourself legally and you do that better together with family members.” [ Physician 10]

So, a patient’s wish for further aggressive treatment and unwillingness to talk about indicated therapy limitation near the end of life can be rooted in family un-readiness of accepting the patient’s situation, resulting in a strong preference for life-prolonging treatment.

### Emotional distress of the family related to the involvement in end-of-life decision-making

An important concern expressed by oncologists and nurses about the family involvement in treatment decisions was that it might overburden them, causing emotional distress, anxiety and depression.:

“I think that relatives are completely overburdened to make such decisions…” [Physician 12], “I think, in general, relatives are shocked even if you prepare them for this […] so that they say do as you consider right…” [Physician 3].

Oncologists deliberately avoided putting family members in the position to decide about treatment intensity since this might generate a psychological burden on them. They also reported that relatives’ emotional proximity to the patient often did not allow them to decide objectively:

“Yes, I think that the family members virtually play a very minor role because relatives would take the initiative and say, he (the patient) shall die now, yes, or we stop now [treatment]…. I think relatives are more likely to cling to the patient like crazy [...] So, I think that family members are completely overwhelmed uh, to make such decisions.” [Physician 12]

### Importance of knowing family structures

Study participants reported that for family involvement in decision-making to limit treatment it is important to know about family structures and interpersonal relationship within a patient’s family: “When the patient is able to make decisions, then, it depends how good the relationship was” [Nurse 4].

And it becomes especially important when patients lose their decisional capacity:

“It depends on how good they understood each other in normal life.” [Nurse 4], “[Family members] I would definitely involve them, yes, and especially if someone is not doing well and can no longer express his will. Yes, but I find that very questionable, when I do not know the family environment.” […] [Physician 4].

When a patient loses his decision-making capacity, the family becomes more involved in the decision-making process as informants about patients’ preferences:

“Um, it's often the case that patients are so weak in this last phase, and no longer have this capacity to decide one hundred percent for themselves how things should be done and then family members play a bigger role.” [Physician 1]

However, participants admitted that it was important to know patients’ family and to determine relatives’ relationship to the patient in order to interpret the agenda put forward by the relatives:

“I think the patient is more important, because there are always situations when the family members pursue certain purposes, yes. But it also depends on how well you know the family situation.” [Physician 4]

All participants acknowledge the importance of knowing family structures, especially by patients who have a limited decision-making capacity.

## Discussion

The statements of the interviewed oncologists and nurses made it clear that they acknowledged the presence of family members and valued their emotional support for patients. However, relatives were also often perceived as second patients who could be traumatized by a patient’s situation and needed special attention, care and time investment.

Participants in our interview study, reported that family members were mostly not involved as long as the patient had the capacity to make decisions; rather, they were seen as informants on patients’ will for incapacitated patients. This corresponds with the legal requirement that involved relatives should not express their own preferences, but should represent and adhere to patients’ authentic wishes and values. According to the patient care legislation in Germany relatives need to be officially designated as a proxy decision-maker either by the patient through an advanced directive or by the court in order to decide on behalf of the patients (§1901a.1 of the German Civil Code (Bürgerliches Gesetzbuch, BGB) [34, 35].

We could identify four certain barriers that might hinder a successful family involvement in end of life decision-making: 1) family impact on patients’ treatment preferences; 2) strong family wish for further treatment; 3) emotional distress of the family related to the involvement in end-of-life decision-making; 4) importance of knowing family structures.

We will discuss them here against the background of the literature and the debate in medical ethics.

### Family impact on patients’ treatment preferences

For the surveyed oncologists and nurses, family members did exert a certain “influence” on patients’ preferences regarding treatment choices. This is in line with other studies. Grunfeld [36] could show i.e. that in up to 28% of patients with breast cancer their relatives were even a key influencing factor in treatment decisions. The interviewees observed in patients with strong family relations an especial a preference for exhausting treatment options in order to gain life-time. This resonates well with our past research that showed that family influence was associated with patients’ striving more for length of life rather than quality of life [37].

Therefore, involving family members in treatment, planning early on, providing them with more information regarding patients’ prognosis and treatment options, could help them to create a realistic view of patients’ situation. Thus, families can even facilitate a transition to the best supportive care. Most patients and families want to discuss forgoing aggressive treatment and the transition to best supportive care with their oncologists and feel frustrated when such discussions do not happen in a timely manner [8].

### Family wish for further treatment

According to the results of our study, family members were occasionally perceived by participants as very demanding if they challenged professional decisions or tried to exert too much influence on medical practice and on the patients. Patients with close family contacts might be prone to life-prolonging treatment in order to comply with family preferences, especially if their relatives insist on further aggressive treatment and deny a patient’s poor prognosis. Hence, there might be a higher risk of disagreement and collusion between relatives and oncologists in decision-making about treatment limitations. It therefore seems absolutely reasonable to involve patients and their relatives early on and to address their treatment expectations in order to prevent situations of disagreement and consecutive ethical and legal conflicts [38]. If family members get the chance to witness the declining effectiveness of therapeutic efforts when the disease is progressing, they can develop a better understanding of the medical complexity and of a limited prognosis of the disease. Studies show that in fact, as patients’ diseases progress, families become more accepting of sharing difficult treatment decisions with the patient and oncologists [39].

In case of very demanding family members some oncologists mention the fear of a legal dispute when they do not comply with the wishes of family members, e.g. when family members request continuation of futile therapy. In that scenario oncologists try to protect themselves from legal action through engaging relatives in proactive conversations.

### Emotional distress of the family related to the involvement in end-of-life decision-making

The interviewees of our study expressed their desire to involve family members, but they also acknowledge that family might suffer from anxiety and depression sometimes even to a greater degree than the patient himself.

Many studies show that family members do suffer from stress, anxiety, fatigue and depression [40–45]. Thus, family members can be hesitant to any involvement in decision-making. In a study by Azoulay et al. half of the family members did not want to participate in treatment decisions about intensive care measures [21]. Others even question the decision-making ability of caregivers in the intensive care setting altogether and doubt the usefulness of involving the partly frightened and traumatised family in the decision-making process at all [46]. For family members of cancer patients, it has been shown that they have unmet needs for support, mainly with regard to fears concerning patients’ condition. Furthermore, they wish to receive a disease-related information and want emotional support for themselves [47]. Family members of cancer patients who were integrated quite early on demonstrated lower depression and distress scores [18]. Hence, family members should be integrated in the decision-making process early on and not only if patients’ condition has worsened in the acute care setting or when end of life decisions have to be made [48, 49]. Furthermore, family members should be perceived as a separate unit from patients with their own needs and wishes as these can considerably differ from that of a patient. Relatives should be offered counselling for social and financial questions and psychological support [50, 51].

### Importance of knowing family structures

In cases of incapacitated patients who cannot express their wishes our interviewed oncologists felt rather unconfident to involve family members, if they did not know them well. This approach is in accordance with a patient care legislation in Germany and the instrument of advanced directives as have been mentioned early in this paper.

Just as the implementation of advanced directives at the beginning of care seemed quite promising as a source for the patients will further clinical practice could show that physicians commonly rely on the family members as substitute decision makers in incapacitated patients [35, 52]. In clinical practice, physicians are often consulted either to interpret the meaning of preferences expressed in patients’ advanced directive or to explore patients’ presumed preferences if no written advanced directive exists. Here, an early contact and exploration of the important decision makers in the family could help oncologists to gain more insight into family matters and potentially conflicting perspectives regarding treatment goals. Insights like these might help oncologists to build consensus between patients and relatives in case of a disagreement. Moreover, any statement of relatives – especially when the patient cannot communicate anymore – need to be understood well against the background of their context, which is often unclear to the observer and, thus, require interpretation. We could not find any existing research which covers this new aspect.

### Limitations of the study

One limitation of this study was that we could not return the interpreted data to participants to check for accuracy and to see if themes were worked out adequately and reflect the participants’ experience due to the lack of time resources of oncologists and nurses.

Our findings are not necessarily transferrable to other care settings. We searched for a maximum variation in our sampling in order to increase the transferability. Our interviews were conducted in a big university hospital that is characterized through a high staff rotation and a considerable time pressure on oncologists and nurses. So, the results might be representative only for university hospitals.

In order to generalize these findings to all hospital settings further quantitative, and complementary qualitative research involving other hospitals and geographic settings is required.

Hence, this qualitative study is a first step towards an understanding of family involvement in decision-making to limit treatment near the end of life and more needs to be done (i.e. investigating different settings, using different research methods) to get a deeper insight into such a complex phenomenon.

## Conclusion

The findings of our study address the existing gap in the literature on the role of patients’ family in decision-making to limit treatment near the end of life when patients are able to make decisions and to communicate with their oncologists and nurses. Although, participants recognised the important role of the family in decision-making, they also acknowledged certain challenges such as possible psychological burden of the relatives, the challenge of knowing family structures, and a strong family wish for further treatment that would seem to account for the reluctance of oncologists for a proactive position regarding family involvement in decision-making.

Strong reasons exist for involving patients’ family early on (if patient authorisation of family involvement is provided) in order to help them to cope with the situation as well as to empower and prepare them for the task to act as surrogates and co-helpers. Not only is early involvement of relatives often in accordance with patient preferences; it also allows for a better understanding of eventual disagreement regarding treatment decisions. Moreover, early integration could assist family members to gain a better grasp of futile therapy situations so that they might be less inclined to insist on non-beneficial anti-cancer treatment or life-sustaining measures for their dying loved-one [48]. The inclusion of additional consultation services such as clinical ethics, pastoral care, psycho-oncology, and clinical social service together with the family, might offer patients and relatives an important aid in processing and mastering disease-specific problems. However, some requirements need to be implemented before early integration is initiated: obtaining the approval for family involvement by the patient, and developing and establishing a systematic assessment of family members’ needs and their wishes in order to provide specific-tailored support.

## Abbreviations

BSC: Best supportive care
ICU: Intensive Care Unit
